# Research of *Panax* spp. in Kunming Institute of Botany, CAS

**DOI:** 10.1007/s13659-018-0176-8

**Published:** 2018-07-06

**Authors:** Yi-Jun Qiao, Jia-Huan Shang, Dong Wang, Hong-Tao Zhu, Chong-Ren Yang, Ying-Jun Zhang

**Affiliations:** 1 State Key Laboratory of Phytochemistry and Plant Resources in West China, Kunming Institute of Botany, Chinese Academy of Sciences, Kunming, 650204 People’s Republic of China; 2 University of Chinese Academy of Sciences, Beijing, 100049 People’s Republic of China; 3 Yunnan Key Laboratory of Natural Medicinal Chemistry, Kunming Institute of Botany, Chinese Academy of Sciences, Kunming, 650201 People’s Republic of China

**Keywords:** *Panax* spp., Phytochemistry, Pharmacological activities, Saponins

## Abstract

**Abstract:**

*Panax*, a genus of the Araliaceae family, is an important herbal group in traditional Chinese medicine (TCM). Nine species and three varieties are included in the genus of *Panax*, in which nearly all species have been used for medicinal purposes. Among them, *Panax notoginseng* (Burk) F. H. Chen, *Panax ginseng* C. A. Meyer and *Panax quinquefolius* L. are the most representative and valuable herbs world-wide, with a long history of cultivation. As the main bioactive chemical constituents, saponins with different aglycones are the major components in various *Panax* spp., and their pharmacological activities are mainly reflected in the effects on blood system, cardio- and cerebro-vascular systems, nervous system, metabolism, and immune regulation. Researchers of Kunming Institute of Botany (KIB), Chinese Academy of Sciences (CAS), have put many efforts into conducting the investigations on *Panax* species. Herein, we reviewed the research progress on *Panax* spp. in KIB, CAS, over the past few decades, from the aspects of history and origin, phytochemistry and pharmacological activities.

**Graphical Abstract:**

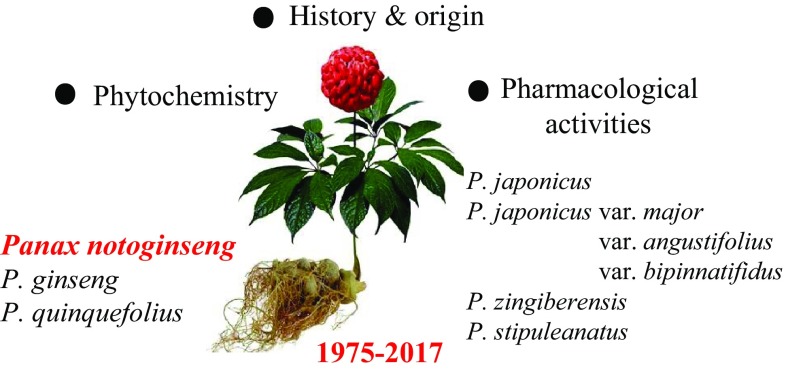

## Introduction

There are 12 species and varieties in *Panax* genus of Araliaceae family all around the world. Six species and three varieties are originated from China, while *P. quinquefolius* L. and *P. trifolium* L. are from North America, and *P. vietnamensis* Ha et Grushv is from Southeast Asia [1]. With extremely high medicinal and economic values, three of the species, e.g., *P. notoginseng* (Burk) F. H. Chen, *P. ginseng* C. A. Meyer and *P. quinquefolius* L. have already become publicly recognized valuable medicinal and edible resources, while many other species from this genus have also been widely used in traditional Chinese medicine (TCM) or folk medicine [2].

As the main bioactive constituents, saponins in different *Panax* spp. with different contents are existed with similar aglycones like panaxadiol, panaxatriol and oleanolic acid [3]. Most *Panax* spp. have often been used medicinally as nourishing drugs for the treatment of bruising, bleeding and muscle pain. The pharmacological activities are mainly reflected in the effects on the blood system, cardiovascular system, cerebrovascular system, nervous system, metabolism, and immune regulation [4, 5].

**Fig. 1:**
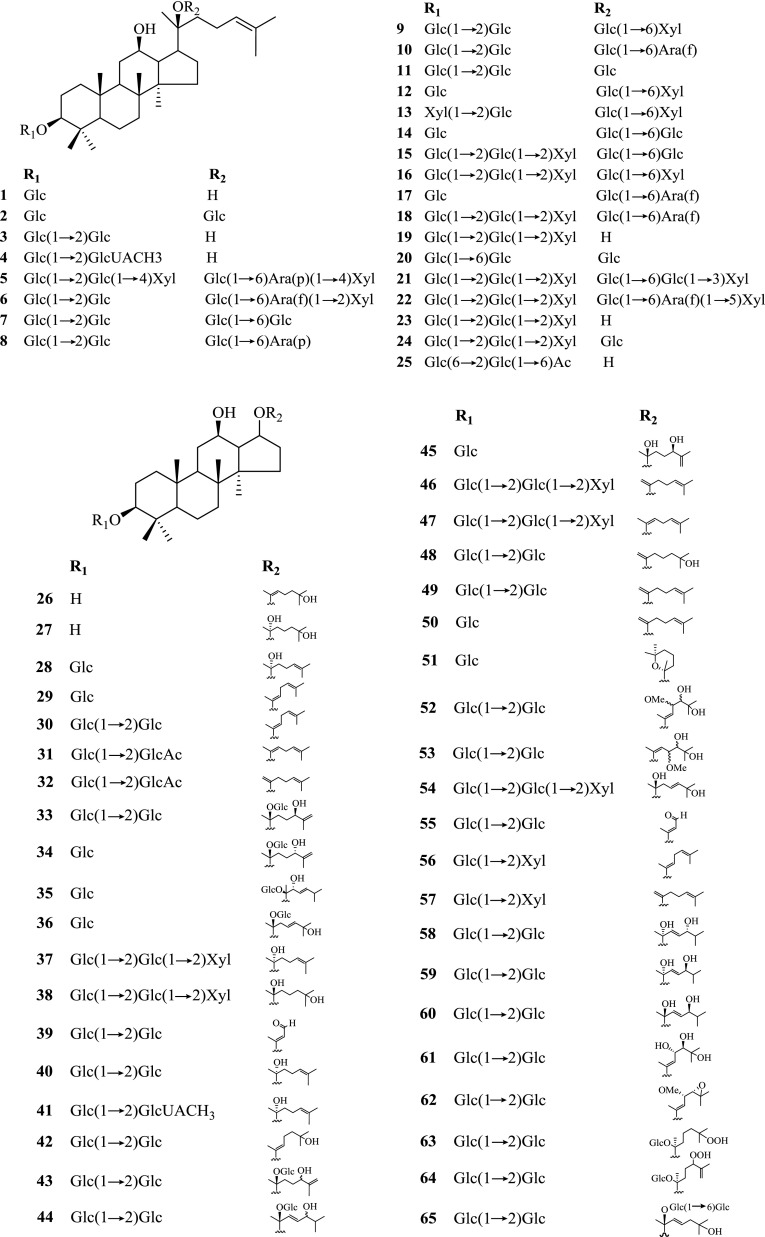

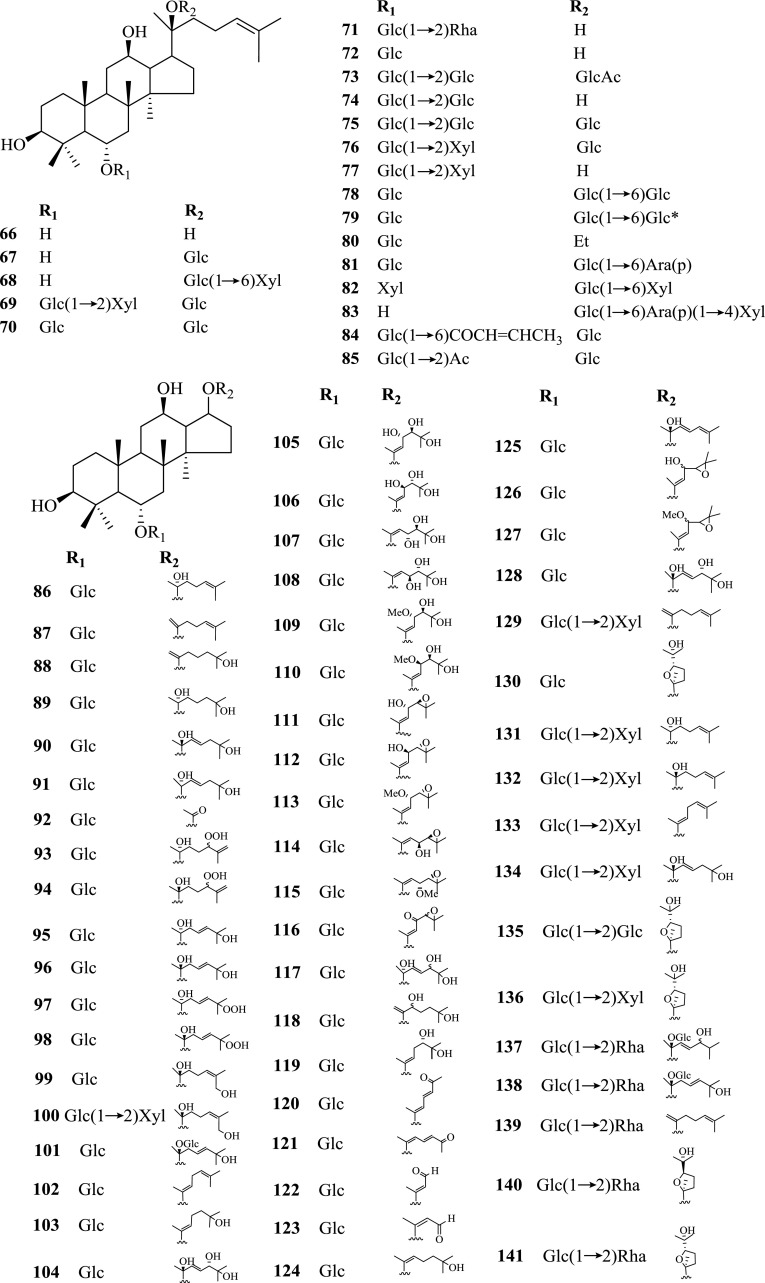

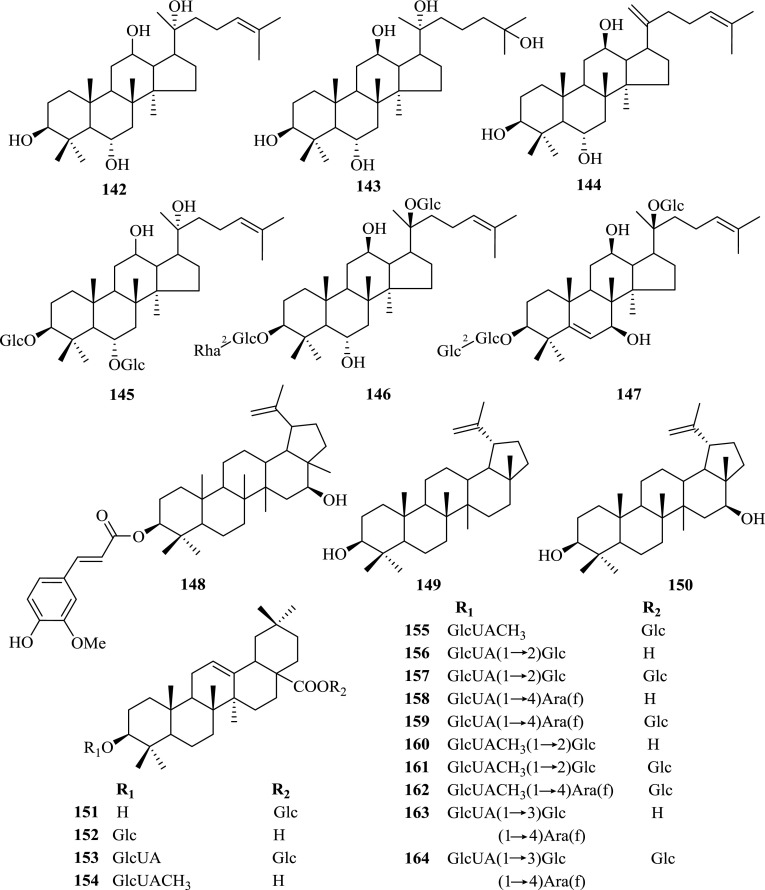
Saponins and their aglycones **1**–**164** from *Panax* spp. Glc, *β*-d-glucose; Glc*, *a*-d-glucose

**Fig. 2:**
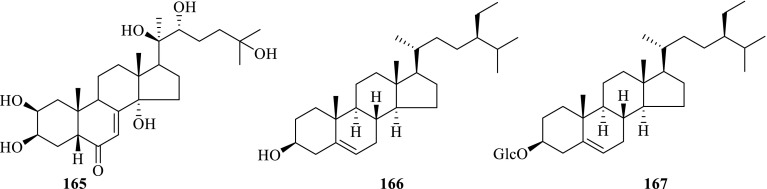
Steroids and their glycosides **165**–**167** from *Panax* spp

**Fig. 3:**
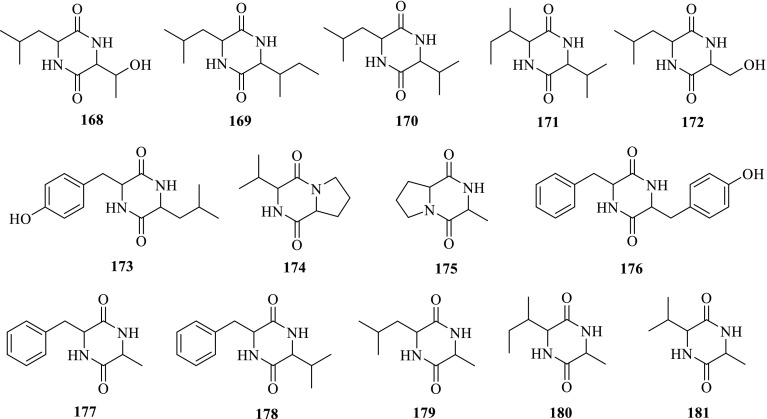
Cyclodipeptides **168**–**181** from *Panax* spp

**Fig. 4:**
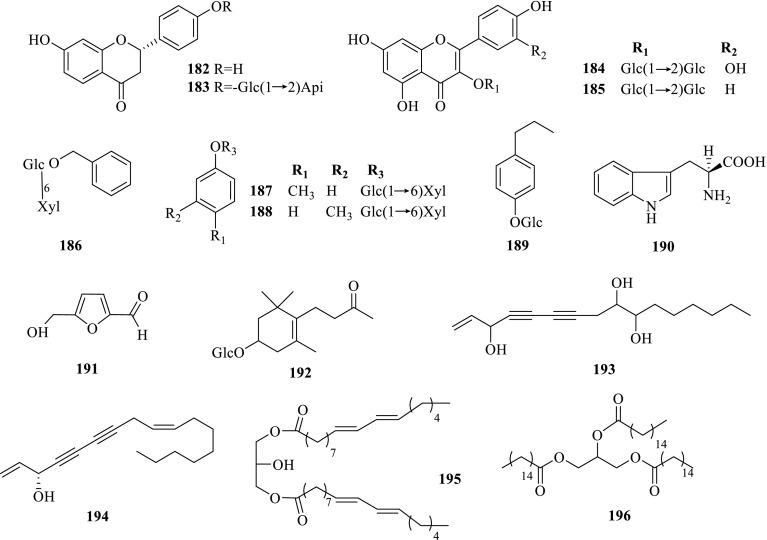
Others **182**–**196** from *Panax* spp

With the contribution from many research groups, investigations on *Panax* spp. in Kunming Institute of Botany (KIB), Chinese Academy of Sciences (CAS) have been lasted for nearly 60 years, leading to the isolation and identification of nearly 200 chemical constituents (Table 1, Figs. 1, 2, 3 and 4), whose pharmacological activities were also studied.

**Table 1:** Chemical constituents of *Panax* spp. and their plant sources

No.	Components	Plant sources	Parts of the plant	Refs.
Saponins and their aglycones				
**1**	20(*S*)-Ginsenoside Rh_2_	*P. notoginseng*	Leaves (hydrolysate), steamed roots, steamed leaves	[27, 34, 35]
**2**	Ginsenoside F_2_	*P. notoginseng*	Flower buds, leaves, fruit pedicels, steamed leaves, rhizomes	[14, 20, 22, 23, 34]
		*P. japonicus* var. *major*	Leaves	[64]
		*P. japonicus* var*. bipinnatifidus*	Leaves	[70]
**3**	20(*S*)-Ginsenoside Rg_3_	*P. notoginseng*	Leaves, leaves (hydrolysate), steamed roots, steamed leaves	[22, 27, 33–35]
			Rhizomes, fibrous biotransformation	[16, 40]
		*P. ginseng*	Roots	[55]
		*P. japonicus* var. *major*	Rhizomes	[63]
		*P. japonicus* var. *bipinnatifidus*	Rhizomes	[69]
**4**	20(*S*)-6″-*O*-Acetylginsenoside Rg_3_	*P. notoginseng*	Steamed roots	[35]
**5**	Ginsenoside Ra_1_	*P. ginseng*	Roots	[55]
**6**	Ginsenoside Ra_2_	*P. ginseng*	Roots	[55]
**7**	Ginsenoside Rb_1_	*P. notoginseng*	Basal part of stems, flower buds, leaves and seeds, leaves	[12, 20–22]
			Fruit pedicels, steamed roots, rhizomes	[15, 16, 23, 33]
		*P. ginseng*	Roots	[55]
		*P. quinquefolium*	Roots	[57]
		*P. japonicus*	Rhizomes	[60]
		*P. japonicus* var. *bipinnatifidus*	Rhizomes, leaves	[69, 70]
**8**	Ginsenoside Rb_2_	*P. notoginseng*	Roots, flower buds, fruit pedicels	[9, 20, 23]
		*P. ginseng*	Roots	[55]
		*P. quinquefolium*	Roots	[57]
**9**	Ginsenoside Rb_3_	*P. notoginseng*	Leaves, seeds, fruit pedicels, steamed leaves	[21–23, 34]
		*P. japonicus* var*. bipinnatifidus*	Leaves	[70]
**10**	Ginsenoside Rc	*P. notoginseng*	Flower buds, leaves, seeds, fruit pedicels	[20–23]
		*P. ginseng*	Roots	[55]
		*P. quinquefolium*	Roots	[57]
**11**	Ginsenoside Rd	*P. notoginseng*	Basal part of stems, flower buds, seeds, leaves, fruit pedicels	[12, 20–23]
			Steamed roots, rhizomes	[15, 16, 33]
		*P. ginseng*	Roots	[55]
		*P. quinquefolium*	Roots	[57]
		*P. japonicus*	Rhizomes	[59, 60]
		*P. japonicus* var*. major*	Rhizomes, leaves	[61, 62, 64]
		*P. zingiberensis*	Rhizomes	[66]
		*P. japonicus* var. *angustifolius*	Rhizomes	[67]
		*P. japonicus* var*. bipinnatifidus*	Rhizomes, leaves	[69, 70]
**12**	Gypenoside IX	*P. notoginseng*	Leaves and seeds, fruit pedicels, steamed leaves	[21, 23, 34]
**13**	Gypenoside XIII	*P. notoginseng*	Leaves, fruit pedicels	[22, 23]
**14**	Gypenoside XVII	*P. notoginseng*	Leaves, fruit pedicels	[22, 23]
		*P. japonicus*	Rhizomes	[60]
**15**	Notoginsenoside Fa	*P. notoginseng*	Leaves, seeds, fruit pedicels, rhizomes	[16, 21–23]
**16**	Notoginsenoside Fc	*P. notoginseng*	Leaves and seeds, fruit pedicels	[21, 23]
**17**	Notoginsenoside Fe	*P. notoginseng*	Leaves	[21]
**18**	Notoginsenoside Fp_2_	*P. notoginseng*	Fruit pedicels	[23]
**19**	20(*S*)-Notoginsenoside Ft_1_	*P. notoginseng*	Steamed leaves	[34]
**20**	Notoginsenoside K	*P. quinquefolium*	Roots	[57]
**21**	Notoginsenoside T	*P. notoginseng*	Rhizomes	[15, 16]
**22**	Notoginsenoside S	*P. notoginseng*	Rhizomes	[15, 16]
**23**	Notoginsenoside R_4_	*P. notoginseng*	Roots, basal part of stems	[11, 12]
**24**	Vina-ginsenoside R_7_	*P. notoginseng*	Fruit pedicels	[23]
**25**	Ginsenoside Rs_3_	*P. notoginseng*	Steamed leaves	[34]
**26**	Dammar-20(22)en-3*β*,12*β*,26-triol	*P. notoginseng*	Leaves (hydrolysate)	[29]
**27**	20(*R*)-Dammaran-3*β*,12*β*,20,25-tetriol	*P. notoginseng*	Leaves (hydrolysate)	[29]
**28**	20(*R*)-Ginsenoside Rh_2_	*P. notoginseng*	Steamed roots, steamed leaves	[34, 35]
**29**	Ginsenoside Rh_3_	*P. notoginseng*	Steamed roots, steamed leaves	[34, 35]
**30**	Ginsenoside Rg_5_	*P. notoginseng*	Roots (hydrolysate), steamed roots, steamed leaves	[26, 33–35]
**31**	Ginsenoside Rs_4_	*P. notoginseng*	Steamed leaves	[34]
**32**	Ginsenoside Rs_5_	*P. notoginseng*	Steamed leaves	[34]
**33**	Majonoside F_1_	*P. japonicus* var. *bipinnatifidus*	Leaves	[70]
**34**	Majonoside F_2_	*P. japonicus* var*. major*	Leaves	[64]
**35**	Majonoside F_3_	*P. japonicus* var*. major*	Leaves	[64]
**36**	Majonoside F_4_	*P. japonicus* var*. major*	Leaves	[64]
**37**	20(*R*)-Notoginsenoside Ft_1_	*P. notoginseng*	Leaves (hydrolysate), steamed leaves	[27, 34]
**38**	Notoginsenoside Ft_2_	*P. notoginseng*	Leaves (hydrolysate)	[27]
**39**	Notoginsenoside Ft_3_	*P. notoginseng*	Leaves (hydrolysate)	[27]
**40**	20(*R*)-Ginsenoside Rg_3_	*P. notoginseng*	Leaves (hydrolysate), steamed roots, steamed leaves, rhizomes	[16, 27, 33–35]
**41**	20(*R*)-6″-*O*-Acetylginsenoside Rg_3_	*P. notoginseng*	Steamed roots	[35]
**42**	25-hydroxyl-(*E*)-20(22)-ene-Ginsenoside Rg_3_	*P. notoginseng*	Steamed roots	[36]
**43**	Bipinnatifidusoside F_1_	*P. japonicus* var. *bipinnatifidus*	Leaves	[70]
**44**	Bipinnatifidusoside F_2_	*P. japonicus* var*. bipinnatifidus*	Leaves	[70]
**45**	Notoginsenoside SFt1	*P. notoginseng*	Steamed leaves	[34]
**46**	Notoginsenoside SFt3	*P. notoginseng*	Steamed leaves	[34]
**47**	Notoginsenoside SFt4	*P. notoginseng*	Steamed leaves	[34]
**48**	25-hydroxyginsenoside Rk_1_	*P. notoginseng*	Steamed roots	[36]
**49**	Ginsenoside Rk_1_	*P. notoginseng*	Steamed roots, steamed leaves	[33–35]
**50**	Ginsenoside Rk_2_	*P. notoginseng*	Steamed roots, steamed leaves	[34, 35]
**51**	Notoginsenoside R_7_	*P. notoginseng*	Roots	[17]
**52**	Notoginsenoside ST-2	*P. notoginseng*	Steamed roots	[33]
**53**	Notoginsenoside ST-3	*P. notoginseng*	Steamed roots	[33]
**54**	Notoginsenoside ST-5	*P. notoginseng*	Steamed roots	[33]
**55**	Notoginsenoside ST-10	*P. notoginseng*	Steamed roots	[36]
**56**	Notoginsenoside ST-11	*P. notoginseng*	Steamed roots	[36]
**57**	Notoginsenoside ST-12	*P. notoginseng*	Steamed roots	[36]
**58**	Notoginsenoside SP_1_	*P. notoginseng*	Steamed roots	[35]
**59**	Notoginsenoside SP_2_	*P. notoginseng*	Steamed roots	[35]
**60**	Notoginsenoside SP_3_	*P. notoginseng*	Steamed roots	[35]
**61**	Notoginsenoside SP_11_	*P. notoginseng*	Steamed roots	[35]
**62**	Notoginsenoside SP_17_	*P. notoginseng*	Steamed roots	[35]
**63**	Notoginsenoside E	*P. notoginseng*	Rhizomes	[14]
**64**	Ginsenoside II	*P. notoginseng*	Rhizomes	[14]
**65**	Koryoginsenoside R_2_	*P. ginseng*	Roots	[55]
**66**	20(*S*)-Protopanaxatriol	*P. notoginseng*	Steamed roots, steamed leaves	[33, 34]
**67**	Ginsenoside F_1_	*P. notoginseng*	Fruit pedicels, rhizomes	[14, 23]
		*P. japonicus* var. *bipinnatifidus*	Leaves	[70]
**68**	Ginsenoside F_3_	*P. japonicus* var. *bipinnatifidus*	Leaves	[70]
**69**	Notoginsenoside J	*P. japonicus*	Rhizomes	[60]
**70**	Ginsenoside Rg_1_	*P. notoginseng*	Roots, basal part of stems, leaves, fruit pedicels,	[9, 12, 22, 23]
			Steamed roots, rhizomes, roots (hydrolysate)	[16, 31, 33]
		*P. ginseng*	Roots	[55]
		*P. quinquefolium*	Roots	[57]
		*P. japonicus*	Rhizomes	[59, 60]
		*P. japonicus var. major*	Leaves	[64]
		*P. zingiberensis*	Rhizomes	[66]
		*P. japonicus var. angustifolius*	Rhizomes	[67]
		*P. japonicus var. bipinnatifidus*	Rhizomes	[69]
**71**	Ginsenoside Rg_2_	*P. notoginseng*	Basal part of stems, steamed roots, rhizomes	[12, 16, 33]
			Fibrous biotransformation, roots (hydrolysate)	[31, 40]
		*P. ginseng*	Roots	[55]
		*P. japonicus*	Rhizomes	[59, 60]
**72**	20(*S*)-Ginsenoside Rh_1_	*P. notoginseng*	Basal part of stems, roots (hydrolysate), steamed roots	[11, 31, 33, 35]
		*P. notoginseng*	Steamed leaves, rhizomes, fibrous biotransformation	[15, 16, 34, 40]
		*P. ginseng*	Roots	[55]
		*P. japonicus*	Rhizomes	[60]
		*P. zingiberensis*	Rhizomes	[66]
		*P. japonicus var. angustifolius*	Rhizomes	[67]
**73**	6′′′-*O*-Acetylginsenoside Re	*P. japonicus*	Rhizomes	[60]
**74**	Ginsenoside Rf	*P. notoginseng*	Steamed roots, rhizomes	[16, 33]
		*P. ginseng*	Roots	[55]
		*P. japonicus*	Rhizomes	[60]
**75**	20-*O*-Glucopyranosyl Rf	*P. notoginseng*	Rhizomes	[16]
		*P. japonicus var. major*	Leaves	[62]
**76**	Notoginsenoside R_1_	*P. notoginseng*	Roots, basal part of stems, leaves, fruit pedicels	[10, 12, 22, 23]
			Steamed roots, rhizomes, roots (hydrolysate)	[15, 16, 31, 33]
		*P. ginseng*	Roots	[55]
		*P. japonicus*	Rhizomes	[60]
		*P. zingiberensis*	Rhizomes	[66]
**77**	Notoginsenoside R_2_	*P. notoginseng*	Roots, basal part of stems, steamed roots, rhizomes	[10, 12, 16, 33]
			Fibrous biotransformation, roots (hydrolysate)	[31, 40]
		*P. japonicus*	Rhizomes	[59, 60]
		*P. japonicus var. major*	Leaves	[62]
		*P. japonicus var. major*	Rhizomes	[63]
**78**	Notoginsenoside R_3_	*P. notoginseng*	Roots	[11]
**79**	Notoginsenoside R_6_	*P. notoginseng*	Roots	[11]
**80**	Notoginsenoside T_3_	*P. notoginseng*	Roots (hydrolysate)	[26]
**81**	Notoginsenoside Fp_1_	*P. notoginseng*	Fruit pedicels	[23]
**82**	Notoginsenoside Rw_1_	*P. notoginseng*	Rhizomes	[16]
**83**	Chikusetsusaponin L_5_	*P. notoginseng*	Fruit pedicels	[23]
**84**	Koryoginsenoside R_1_	*P. notoginseng*	Steamed roots, rhizomes	[16, 33]
		*P. ginseng*	Roots	[55]
**85**	Yesanchinoside D	*P. notoginseng*	Steamed roots	[33]
**86**	20(*R*)-Ginsenoside Rh_1_	*P. notoginseng*	Steamed roots, roots (hydrolysate)	[31, 33, 35]
**87**	Ginsenoside Rk_3_	*P. notoginseng*	Steamed roots	[33, 35]
**88**	25-hydroxyginsenoside Rk_3_	*P. notoginseng*	Steamed roots	[35]
**89**	Notoginsenoside SFt_2_	*P. notoginseng*	Steamed roots, steamed leaves, roots (hydrolysate)	[31, 33–35]
**90**	Notoginsenoside R_8_	*P. notoginseng*	Roots	[13]
**91**	Notoginsenoside R_9_	*P. notoginseng*	Roots	[13]
**92**	Notoginsenoside R10	*P. notoginseng*	Steamed roots	[36]
**93**	20(*S*)-Ginsenoside SG_2_	*P. notoginseng*	Steamed roots	[36]
**94**	20(*R*)-Ginsenoside SL_1_	*P. notoginseng*	Steamed roots	[36]
**95**	20(*S*)-Ginsenoside ST_2_	*P. notoginseng*	Steamed roots	[36]
**96**	20(*R*)-Ginsenoside ST_2_	*P. notoginseng*	Steamed roots	[36]
**97**	20(*S*)-Floralquinquenoside A	*P. notoginseng*	Steamed roots	[36]
**98**	20(*R*)-Ginsenoside SF	*P. notoginseng*	Steamed roots	[36]
**99**	Yesanchinoside R_1_	*P. japonicus*	Rhizomes	[60]
**100**	Yesanchinoside R_2_	*P. japonicus*	Rhizomes	[60]
**101**	Vinaginsenoside R_15_	*P. japonicus*	Rhizomes	[60]
**102**	Ginsenoside Rh_4_	*P. notoginseng*	Roots (hydrolysate), steamed roots, rhizomes	[15, 16, 31, 33, 35]
			Fibrous biotransformation, seeds	[24, 40]
**103**	Sanchinoside B_1_	*P. notoginseng*	Steamed roots	[33, 35]
**104**	Notoginsenoside SP_4_	*P. notoginseng*	Steamed roots	[35]
**105**	Notoginsenoside SP_5_	*P. notoginseng*	Steamed roots	[35]
**106**	Notoginsenoside SP_6_	*P. notoginseng*	Steamed roots	[35]
**107**	Notoginsenoside SP_7_	*P. notoginseng*	Steamed roots	[35]
**108**	Notoginsenoside SP_8_	*P. notoginseng*	Steamed roots	[35]
**109**	Notoginsenoside SP_9_	*P. notoginseng*	Steamed roots	[35]
**110**	Notoginsenoside SP_10_	*P. notoginseng*	Steamed roots	[35]
**111**	Notoginsenoside SP_12_	*P. notoginseng*	Steamed roots	[35]
**112**	Notoginsenoside SP_13_	*P. notoginseng*	Steamed roots	[35]
**113**	Notoginsenoside SP_14_	*P. notoginseng*	Steamed roots	[35]
**114**	Notoginsenoside SP_15_	*P. notoginseng*	Steamed roots	[35]
**115**	Notoginsenoside SP_16_	*P. notoginseng*	Steamed roots	[35]
**116**	Notoginsenoside SP_18_	*P. notoginseng*	Steamed roots	[35]
**117**	Notoginsenoside SP_20_	*P. notoginseng*	Steamed roots	[37]
**118**	Notoginsenoside SP_21_	*P. notoginseng*	Steamed roots	[37]
**119**	Notoginsenoside ST_1_	*P. notoginseng*	Steamed roots	[33, 35]
**120**	Notoginsenoside ST_6_	*P. notoginseng*	Steamed roots	[36]
**121**	Notoginsenoside ST_7_	*P. notoginseng*	Steamed roots	[36]
**122**	Notoginsenoside ST_8_	*P. notoginseng*	Steamed roots	[36]
**123**	Notoginsenoside ST_9_	*P. notoginseng*	Steamed roots	[36]
**124**	Notoginsenoside ST_13_	*P. notoginseng*	Steamed roots	[36]
**125**	Notoginsenoside ST_14_	*P. notoginseng*	Steamed roots	[36]
**126**	Notoginsenoside T_1_	*P. notoginseng*	Roots (hydrolysate)	[26]
**127**	Notoginsenoside T_2_	*P. notoginseng*	Roots (hydrolysate)	[26]
**128**	Notoginsenoside T_4_	*P. notoginseng*	Roots (hydrolysate), steamed roots	[26, 35]
**129**	Notoginsenoside T_5_	*P. notoginseng*	Roots (hydrolysate), steamed roots, rhizomes	[14, 16, 26, 36]
**130**	24(*R*)-PseudosingenosideRT_5_	*P. quinquefolium*	Roots	[57]
**131**	20(*S*)-Notoginsenoside R_2_	*P. notoginseng*	Steamed roots	[36]
**132**	20(*R*)-Notoginsenoside R_2_	*P. notoginseng*	Steamed roots	[36]
**133**	3*β*,12*β*-dihydroxydammarane-(*E*)-20(22),24- diene-6-*O*-*β*-D-xylopyranosyl-(1 → 2)-*β*-D- glucopyranoside	*P. notoginseng*	Steamed roots	[36]
**134**	Notoginsenoside Rw_2_	*P. notoginseng*	Rhizomes	[16]
**135**	Majonoside R_1_	*P. japonicus var. major*	Rhizomes, leaves	[61, 62]
**136**	Majonoside R_2_	*P. japonicus* var. *major*	Rhizomes, leaves	[61, 62]
**137**	Majonoside F_5_	*P. japonicus* var. *major*	Leaves	[65]
**138**	Majonoside F_6_	*P. japonicus* var. *major*	Leaves	[65]
**139**	Ginsenoside Rg_6_	*P. notoginseng*	Steamed roots	[36]
**140**	20(*S*)-Pseudoginsenoside F_11_	*P. japonicus* var. *bipinnatifidus*	Rhizomes, leaves	[69, 70]
**141**	20(*R*)-Pseudoginsenoside F_11_	*P. quinquefolium*	Roots	[57]
**142**	20(*R*)-Protopanaxatriol	*P. notoginseng*	Steamed roots, steamed leaves	[33, 34]
**143**	20(*R*)-dammarane-3*β*,6*α*,12*β*,20,25-pentol	*P. notoginseng*	Steamed roots	[35]
**144**	3*β*,6*α*,12*β*-trihydroxydammar-20(21),24-diene	*P. notoginseng*	Steamed leaves	[34]
**145**	3-*O*-*β*-D-glucopyranosyl-6-*O*-*β*-D-glucopyranosyl-20(*S*)-protopanaxatriol	*P. notoginseng*	Roots biotransformation	[39]
**146**	Ginsenoside Re	*P. notoginseng*	Basal part of stems, leaves, fruit pedicels	[12, 22, 23]
		*P. notoginseng*	Steamed roots, rhizomes, roots (hydrolysate)	[15, 16, 23, 31]
		*P. ginseng*	Roots	[55]
		*P. quinquefolium*	Roots	[57]
		*P. japonicus*	Rhizomes	[59, 60]
		*P. japonicus* var. *major*	Rhizomes, leaves	[63, 64]
		*P. japonicus* var. *bipinnatifidus*	Rhizomes, leaves	[69, 70]
**147**	Notoginsenoside G	*P. japonicus*	Rhizomes	[60]
**148**	Lup-2-ene-3*β*,16*β*-diol-3-ferulate	*P. notoginseng*	Seeds	[24]
**149**	Lupeol	*P. notoginseng*	Seeds	[24]
**150**	16*β*-Hydroxy lupeol	*P. notoginseng*	Seeds	[24]
**151**	Oleanolic acid 28-*O*-*β*-D-glucopyranoside	*P. japonicus*	Rhizomes	[60]
		*P. japonicus* var. *major*	Rhizomes	[63]
		*P. japonicus* var*. angustifolius*	Rhizomes	[67]
**152**	Oleanolic acid 3-*O*-*β*-D-glucopyranoside	*P. japonicus* var*. angustifolius*	Rhizomes	[67]
**153**	Chikusetsusaponin IVa	*P. japonicus*	Rhizomes	[59, 60]
		*P. japonicus* var. *major*	Rhizomes, leaves	[61–63]
		*P. zingiberensis*	Rhizomes	[66]
		*P. japonicus* var. *angustifolius*	Rhizomes	[67]
		*P. japonicus* var. *bipinnatifidus*	Rhizomes, leaves	[69, 70]
**154**	3-*O*-*β*-D-(6′-methyl ester) glucuronopyranoside	*P. japonicus*	Rhizomes	[60]
**155**	Chikusetsusaponin IVa methyl ester	*P. japonicus*	Rhizomes	[60]
		*P. japonicus* var. *major*	Rhizomes	[60]
**156**	Zingibroside R_1_	*P. zingiberensis*	Rhizomes	[66]
		*P. japonicus* var*. angustifolius*	Rhizomes	[67]
		*P. japonicus* var. *bipinnatifidus*	Rhizomes	[69]
**157**	Chikusetsusaponin V(ginsenoside R_0_)	*P. ginseng*	Roots	[55]
		*P. japonicus*	Rhizomes	[59, 60]
		*P. japonicus var. major*	Rhizomes, leaves, rhizomes	[61, 62, 65]
		*P. zingiberensis*	Rhizomes	[66]
		*P. japonicus* var*. angustifolius*	Rhizomes	[67]
		*P. japonicus* var. *bipinnatifidus*	Rhizomes, leaves	[69, 70]
**158**	Polysciassaponin P_5_	*P. japonicus*	Rhizomes	[60]
**159**	Chikusetsusaponin IV	*P. japonicus*	Rhizomes	[59, 60]
		*P. zingiberensis*	Rhizomes	[66]
		*P. japonicus* var*. angustifolius*	Rhizomes	[67]
		*P. japonicus* var. *bipinnatifidus*	Rhizomes, leaves	[69, 70]
**160**	Oleanolic acid 3-*O*-*β*-D-glucosyl-(1 → 2)-*β*-D-(6′-methylester)glucuronoside	*P. japonicus*	Rhizomes	[60]
**161**	Chikusetsusaponin V methyl ester	*P. japonicus*	Rhizomes	[60]
**162**	Chikusetsusaponin IV methyl ester	*P. japonicus*	Rhizomes	[60]
**163**	Stipuleanoside R_1_	*P. stipuleanatus*	Rhizomes	[68]
**164**	Stipuleanoside R_2_	*P. stipuleanatus*	Rhizomes	[68]
Steroids and their glycoside				
**165**	Ecdysterone	*P. notoginseng*	Steamed roots	[37]
		*P. japonicus*	Rhizomes	[60]
**166**	*β*-Sitosterol	*P. notoginseng*	Seeds	[24]
**167**	Daucosterol	*P. notoginseng*	Seeds	[24]
		*P. japonicus*	Rhizomes	[60]
Cyclodipeptides				
**168**	Cyclo-(Leu-Thr)	*P. notoginseng*	Roots	[18]
**169**	Cyclo-(Leu-Ile)	*P. notoginseng*	Roots	[18]
**170**	Cyclo-(Leu-Val)	*P. notoginseng*	Roots	[18]
**171**	Cyclo-(Ile-Val)	*P. notoginseng*	Roots	[18]
**172**	Cyclo-(Leu-Ser)	*P. notoginseng*	Roots	[18]
**173**	Cyclo-(Leu-Tyr)	*P. notoginseng*	Roots	[18]
**174**	Cyclo-(Val-Pro)	*P. notoginseng*	Roots	[18]
**175**	Cyclo-(Ala-Pro)	*P. notoginseng*	Roots	[18]
**176**	Cyclo-(Phe-Tyr)	*P. notoginseng*	Roots	[18]
**177**	Cyclo-(Phe-Ala)	*P. notoginseng*	Roots	[18]
**178**	Cyclo-(Phe-Val)	*P. notoginseng*	Roots	[18]
**179**	Cyclo-(Leu-Ala)	*P. notoginseng*	Roots	[18]
**180**	Cyclo-(Ile-Ala)	*P. notoginseng*	Roots	[18]
**181**	Cyclo-(Val-Ala)	*P. notoginseng*	Roots	[18]
Others				
**182**	Liquiritigenin	*P. notoginseng*	Leaves	[22]
**183**	Liquiritin apioside	*P. notoginseng*	Leaves	[22]
**184**	Quercetin 3-*O*-*β*-D-glucopyranosyl-(1 → 2)-*β*-D-galactopyranoside	*P. notoginseng*	Fruit pedicels	[23]
**185**	Kaempferol 3-*O*-*β*-D-glucopyranosyl-(1 → 2)-*β*-D-galactopyranoside	*P. notoginseng*	Fruit pedicels	[23]
**186**	Benzyl-*β*-primeveroside	*P. notoginseng*	Fruit pedicels	[23]
**187**	*p*-methyl phenyl glycosides	*P. notoginseng*	Steamed roots	[37]
**188**	*m*-methyl phenyl glycosides	*P. notoginseng*	Steamed roots	[37]
**189**	*β*-ethylphenyl-1-*O*-*β*-D-glucopyranoside	*P. notoginseng*	Steamed roots	[37]
**190**	(*S*)-Tryptophan	*P. notoginseng*	Fruit pedicels	[23]
**191**	5-hydroxymethyl-2-furancarboxaldehyde	*P. notoginseng*	Steamed roots	[33]
**192**	Icariside B_6_	*P. notoginseng*	Fruit pedicels	[23]
**193**	Panaxytriol	*P. notoginseng*	Roots and steamed roots	[17, 33]
**194**	Panaxynol	*P. notoginseng*	Seeds	[24]
**195**	(*Z,Z*)-9,12-Octadecadienoic acid 2-hydroxy-1,3-propanedinyl ester	*P. notoginseng*	Steamed roots	[33]
**196**	Hexadecanoic acid glycerin ester	*P. notoginseng*	Seeds	[24]

Herein, we reviewed the research work on *Panax* spp. in KIB, CAS, from the aspects of history and origin, phytochemistry and pharmacological activities. Future perspectives in this researching field were also discussed. Among all the species investigated in KIB, studies on *P. notoginseng* accounted for the largest proportion. Thereby, we presented its related works in detail specifically and summarized the studies on other *Panax* spp. (*P. ginseng, P. quinquefolius, P. japonicus, P. japonicus* var. *major, P. zingiberensis, P. japonicus* var. *angustifolius, P. stipuleanatus, P. japonicus* var. *bipinnatifidus*) more briefly as well.

## Research on *Panax notoginseng*

*Panax notoginseng*, one of the earliest cultivated plants in ginseng species, has a cultivation history of more than 400 years in Wenshan, Yunnan Province and Jingxi, Guangxi Province [6]. As a crucial TCM and a long-established natural resource for medicine and food, *P. notoginseng* has been traditionally used as a tonic and hemostatic drug for promoting blood circulation, curing bruises, and treating blood loss caused by internal and external injuries. The main bioactive components in *P. notoginseng* are saponins, which been isolated and identified from different parts of *P. notoginseng*, together with amino acids, polysaccharides, flavonoids, acetylenic alcohols, and volatile oils [3].

Research of *P. notoginseng* can be traced back to the 1930s. Scientific staff in KIB, CAS began their explorations in the 1960s. During the 1980s, under the leadership of Professor J. Zhou, systematic phytochemical investigation on *P. notoginseng* was strengthened, and some of the initial work was conducted with Japanese scholars together. Afterwards, phytochemical and pharmacological investigations of *P. notoginseng* were mainly carried out by Prof. C.R. Yang and Prof. Y. J. Zhang’s research group.

### History and Origin

In 1975, through the comparative study of triterpenoids constituents, taxonomy and geographic distribution of various *Panax* spp., *P. notoginseng*, *P. ginseng* and *P. quinquefolius* were considered as the ancient taxa of Ginseng plant and *P. notoginseng* was suggested to be the oldest member among living species of *Panax* [3].

Based on the ancient literature researches and plant biology investigations, the history of utilization and cultivation of *P. notoginseng* as well as the original places of this herb were discussed by Prof. C. R. Yang in 2015. The paper suggested *P. notoginseng* was first used in ethnic minorities (Miao, Zhuang, Yao and Yi) in the southwest of Guangxi and southeastern Yunnan. With the exchanges among various ethnic groups and the spread of military and merchants, it was gradually introduced into the Central Plains. The effectiveness and role of *P. notoginseng* have been continuously discovered. It has become a well-known expensive drug in the Ming and Qing Dynasties [7].

Further study was carried out in 2017, focused on the record and application of *P. notoginseng* in TCM as well as its development in recent years, throughout the investigation of ancient herbs and herbal prescriptions, the history of the use and dissemination of *P. notoginseng* in China were verified, with the source, dissemination, distribution of origin and its marketing trade analyzed together [8].

### Phytochemistry

Saponins were characterized as the major type of compounds in *P. notoginseng*, together with other minor constituents such as cyclodipeptides, flavonoids, sterols and polyacetylenes. Summarized totally as 159 of them, their structures were shown below (Figs. 1, 2, 3 and 4), with their names and the corresponding plant sources organized together in Table 1.

#### Saponins

As one of the main bioactive components in medicinal plants of *Panax* spp., saponins were found to dominate the chemical composition of *P. notoginseng*.

For the past decades, large quantities of saponins were isolated and identified from the underground and aboveground parts as well as the cell cultures of *P. notoginseng* [3, 9–25]. These saponins could all be divided into two groups, either 20(*S*)-protopanaxadiol or 20(*S*)-protopanaxa- triol, which were referred to as the Rb-group and Rg-group saponins respectively. With the same nucleus, these dammarane-type tetracyclic triterpenoid saponins possess a variety of aglycones and glycosyl groups with different structures.

Besides, several transformation processes were conducted, with chemical, physical or biological method, large amounts of transformed products were obtained, and some of them were proved to be bioactive.

For example, under the circumstance of mild acid hydrolysis, eight new dammarane-type saponins were isolated from the hydrolyzed products of total saponins of *P. notoginseng*, named as notoginsenoside T_1_-T_5_ (**126, 127, 80, 128, 129**) [26], (20*S/R*)-notoginsenoside Ft_1_ (**19, 37**) and notoginsenoside Ft_2_-Ft_3_ (**38, 39**) [27]. While a series of secondary saponins and glycosides deglycosylated at C-20 position were obtained from hydrolysates of ginsenoside and notoginsenoside [28–31].

As early as 1985, the saponins of raw and steamed *P. notoginseng* were compared. It was found that the yield of bisglycosyl saponins was decreased and the monosaccharide saponins was increased after processing of steam, indicating that the dammarane-type saponins were not stable and could be degraded at a high temperature [32]. After 2000, the chemical constituents of steamed *P. notoginseng* was studied systematically, 96 dammarane saponins were isolated and purified from steamed roots, rhizomes and leaves of *P. notoginseng* [32–36]. Meanwhile, some were found to have the inhibitory activity of acetylcholinesterase and the activity of promoting the differentiation of PC12 cells [35–37]. The dynamic changes of saponins under different transformation conditions, the effects of different factors on saponins’ transformation and the ways to transform saponins were preliminarily discussed as well [38].

Then by using biotransformation method, study on the fermentation of saponins from *P. notoginseng* with *Bacillus subtilis* led to the isolation of ginsenoside Rh_4_ (**102**), which hadn’t been reported or detected in the raw material of *P. notoginseng* by that time. Ginsenoside Rh_1_ (**72**) was also biotransformed by *B. subtilis*, yielding a new triterpene saponin, 3-*O*-*β*-d-glucopyranosyl-6-*O*-*β*-d-glucopyranosyl-20(*S*)-protopanaxatriol (**145**) [39, 40].

As for qualitative and quantitative analysis, saponins in the underground parts of *P. notoginseng* were analyzed and the contents of five main saponins, ginsenoside Rg_1_ (**70**), Rb_1_ (**7**), Re (146), Rd (**11**) and notoginsenoside R_1_ (**76**) were compared. The results showed that the contents of ginsenosides Rg_1_ and Rb_1_, together with total contents of the five main saponins in the taproot “60 Tou” (viz. 60 taproots per 500 g) were highest among all commercial grades of *P. notoginseng*. With only around 18% biomass of the underground parts, the rhizome provided more than 25% saponins. The levels of biomass and saponins of phloem in both taproot and rhizome are significantly higher than those of xylem. Besides, the biomass and saponin levels of 2-year-old roots are markedly lower than those of 3-year-old ones. The comparative analyses were also carried out on *P. notoginseng* of different stem colors [41]. Furthermore, by studying the chemical compositions, morphological differences and the relationships between individuals of *P. notoginseng*, it was found that great differences exist in content, distribution and variation of total saponins, proportion of each component and morphological characteristics [42, 43]. In addition, the formation and accumulation of saponins in *P. notoginseng* roots during germination and juvenile stage were investigated. As the results showed, the chemical composition of seed was found greatly different from that of root and there was little saponin in the seed of *P. notoginseng*. The accumulation of saponins, which was affected by seasons, showed a time-dependent increase after germination of *P. notoginseng* [44].

From another aspect, the effects of oligosaccharins of *D. candidum* (DO), *P. ginseng* (GO) and *C. tinctoris* (CO) on callus growth and saponin content of *P. notoginseng* were also investigated. The results showed that with appropriate concentration, all of the three kinds of biologically active and wall-related oligosaccharins could stimulate saponin formation or callus growth, which provide a possibly good way to produce saponin by using oligosaccharins in large scale culture [45].

Then from 2000 to 2002, the ^1^H and ^1^C chemical shifts of protopanaxadiol-type mono- desmosidic ginsenoside Rg_5_ (**30**), (20*S/R*)-ginsenoside Rg_3_ (**3**, **40**) [46], ginsenoside Rd (**11**), notoginsenoside E (**63**) and gypenoside XVII (**14**) [47] were fully specified respectively, by using 2D-NMR techniques for the first time.

Except for the chemical sequencing routine, efforts have been put into genetical research as well. Genetic diversity and variation of saponin contents between individual *P. notoginseng* roots harvested from a single location were tested by chemical analysis and DNA fingerprinting. High- performance TLC together with HPLC analysis were used to analyze the presence of six saponins (ginsenoside Rb_1_, Rg_1_, Rd, Re and Rc, notoginsenoside R_1_). The samples were also subjected to fluorescent amplified fragment length polymorphism (AFLP) analysis, and their internal transcribed spacer 2 (ITS 2) regions of the samples were sequenced. In conclusion, genetic diversity and variation of saponin contents between individual *P. notoginseng* roots have been detected and genetic factors may play a leading role in causing chemical differences, such as affecting the contents of the six saponins mentioned above in *P. notoginseng*, while environment is the secondary influential factor [48].

#### Cyclodipeptides

In 2004, 14 cyclodipeptides including one new compound, and seven new natural products were isolated from the roots of *P. notoginseng* by Prof. N.H. Tan’s research group. They were identified by spectral methods, namely cyclo-(Leu-Thr) (**168**), cyclo-(Leu-Ile) (**169**), cyclo-(Leu-Val) (**170**), cyclo-(Ile-Val) (**171**), cyclo-(Leu-Ser) (**172**), cyclo-(Leu-Tyr) (**173**), cyclo-(Val-Pro) (**174**), cyclo-(Ala-Pro) (**175**), cyclo-(Phe-Tyr) (**176**), cyclo-(Phe-Ala) (**177**), cyclo-(Phe-Val) (**178**), cyclo-(Leu-Ala) (**179**), cyclo-(Ile-Ala) (**180**) and cyclo-(Val-Ala) (**181**). Among them Compounds cyclo-(Leu-Ile) (**169**) and cyclo-(Phe-Val) (**178**), cyclo-(Leu-Val) (**170**) and cyclo-(Ile-Val) (**171**), cyclo-(Leu-Ala) (**179**) and cyclo-(Phe-Val) (**178**) are mixtures with 2:1, 1:1 and 2:1 ratios, respectively [18].

#### Others

Many other kinds of natural products such as flavonoids, phenolic glycosides, alkynols, amino acid, esters, furfural and *O*-Glycoside et al. have been investigated as well. Among which, phenolic glycosides, furfural and *O*-Glycoside were isolated from steamed roots of *P. notoginseng* [33, 37], with alkynols from roots [17], flavonoids and phenolic glycosides from leaves [22], flavonoids, phenolic glycosides, amino acid and *O*-Glycoside from fruit pedicels [23], and alkynols and esters from seeds as well [24].

### Pharmacological Activities

For the past few years, in comparison with pharmacology, much more effort has been put into phytochemistry in research of *Panax* spp. in KIB. Even though, the chemical research work provided a basis for the study on pharmacological activities of compounds yielded from plants in the genus of *Panax*, and some of the bioactive compounds have been detected and selected from large quantities of natural products.

Notoginseng Radix et Rhizoma has the efficacy of dissolving stasis and hemostasis and reducing swelling and easing pain. *P. notoginseng* saponins (PNS) is the main active component of Notoginseng Radix et Rhizoma, and the main components include ginsenoside Rb_1_ (**7**), Rg_1_ (**70**), Re (**146**), Rd (**11**) and notoginsenoside R_1_ (**76**), which were proved to contribute to several pharmacological activities of *P. notoginseng* in the blood system, cardiovascular system, cerebrovascular system, nervous system and so on.

#### Antithrombotic Effect

In 2002, it was found that ginsenoside Rg_1_ (**70**) had a strong antithrombotic effect which can prolong the thrombotic time by significantly inhibiting the adhesion of neutrophil to thrombin-stimulated platelets. Charlton and Rosette test were used to evaluate the effect of ginsenoside Rg_1_ on carotid thrombosis induced by electrical stimulation and to observe its effect on the adhesion of neutrophil to platelet in rat respectively [49].

#### Effects on DNA and Protein Metabolism

Total saponins of *P. notoginseng* (PNS) was proved to have a positive effect on the synthesis of DNA and protein in mice poisoned by carbon tetrachloride. According to the experiment results, PNS can promote the corporation rate of ^3^H-TdR to DNA and ^3^H-leucine to liver and serum protein on hepatic injury in mice. Microscopic examination also showed that hepatocellular proliferation in PNS group was significantly greater than that in the control group. These experimental results show that PNS has a certain role in promoting liver regeneration in CCl_4_ liver-injured mice from different aspects [50].

#### Effects on the Cardiovascular System

In 2017, Song et al. reviewed the research progress in pharmacological effects, clinical application and adverse reactions of PNS in treatment of cerebral vascular disease [51]. It suggested that PNS played an important and complex role in curing cerebrovascular diseases, with effects like inhibiting platelet aggregation, antithrombosis, reducing blood viscosity, increasing tissue blood flow, improving microcirculation and energy metabolism, blocking calcium channels and reducing cerebral edema, protecting brain and heart muscle, as well as anti-arrhythmia and shock, etc. [52–54].

## Research on Other *Panax* spp

### *Panax ginseng* C. A. Meyer

*Panax ginseng*, a perennial herb of *Panax* spp. in the Araliaceae family, is a precious resource for traditional Chinese medicine, known as “the king of herbs.” It is distributed and cultivated mainly in Northeast of China, partially in Russia and North Korea, which have also been introduced into cultivation in Hebei and Shanxi province in China, as well as Japan. Located in the eastern part of Liaoning, Jilin and Heilongjiang, it is found in deciduous broad-leaved forests or coniferous and broad-leaved mixed forests several hundred meters above sea level.

Historically Chinese have been taken *P. ginseng* as a natural invigorant in nourishing and strengthening life, which was supposed to reinforce vital energy, adjust blood pressure, restore heart function and physical weakness, promote the secretion of saliva or body fluid, and calm the nerves [5]. Tetracyclic triterpenoids of dammarane type are the main constituents in *P. ginseng*, which have been proved to possess lots of pharmacological activities [1].

In 1995, Korean scholar D. S. Kim, guided by Professor C. R. Yang, isolated and identified two new minor dammarane saponins named Koryoginsenoside R_1_ (**84**) and R_2_ (**65**), along with 14 known saponins, namely ginsenoside R_0_ (**157**), Ra_1_ (**5**), Ra_2_ (**6**), Rb_1_ (**7**), Rb_2_ (**8**), Rc (**10**), Rd (**11**), Rg_3_ (**3**), Re (**146**), Rf (**74**), Rg_1_ (**70**), Rg_2_ (**71**), Rh_1_ (**72**) and notoginsenoside R_1_ (**76**) [55].

### *Panax quinquefolius* L

*Panax quinquefolius* is a plant of the genus of *Panax*, which is originated from North America. It’s morphology is very similar to *P. ginseng*, and has been cultivated in the same areas of *P. ginseng* in China for so many years. As a medicinal herb, it is often used to clear heat, cure chronic lung disease with cough, blood loss, throat thirst, and irritability [3].

In 1989, Yang et al. analyzed the composition and contents of *P. quinquefolium* cultivated in Yunnan, China, by high-performance liquid chromatography (HPLC). They also differentiated the contents of the major saponins including ginsenoside Rb_1_ (**7**), Rb_2_ (**8**), Rc (**10**), Rd (**11**), Re (**146**), Rg_1_ (**70**), R_0_ (**157**) and malonyl saponins (malonyl ginsenoside Rb_1_, Rb_2_ and Rc) according to the age, time of harvest, commercial grades and the underground parts of the plant [56].

In 2003, 10 saponins, named as 24(*R*)-pseudoginsenoside RT_5_ (**130**), F_11_ (**141**), ginsenoside Rg_1_ (**70**), Re (**146**), Rd (**11**), Rc (**10**), Rb_1_ (**7**), Rb_2_ (**8**), 24(*R*)-ginsenoside Rg_3_ (**40**) and notoginsenoside K (**20**) were isolated and identified from *P. quinquefolium* cultivated in Jilin province of China. Among them, 24(*R*)-pseudoginsenoside RT_5_ (**130**) was isolated from this plant for the first time [57]. To control the quality of American Ginseng, HPLC was carried out on *P. quinquefolius* cultivated in Vancouver, Toronto, Beijing, Shandong and Jilin. Distinct differences were found among American Ginseng produced in different places through quantitative analysis and PCA [58].

### *Panax japonicus* C. A. Meyer

*Panax japonicus*, with the Chinese name “Zhu-Jie-Shen”, belongs to the genus of *Panax*. The rhizome is recorded in the Chinese Pharmacopoeia and used to enhance immunity, diminish inflammation, and transform phlegm [2]. It is also cultivated and used as a medicinal herb in Japan, Korea, and Europe for the treatment of lifestyle-related diseases, such as alcohol-induced gastric ulcer and high-fat-diet-induced obesity. Oleanane- and dammarane-type triterpenoid saponins were reported to be the characteristic components of this herb [60].

In 1983, C. R. Yang along with Japanese researchers isolated oleanane-type saponins chikusetsusaponin IV (**159**), IVa (**153**), V (**157**) and dammarane-type saponins ginsenoside Rd (**11**), Re (**146**), Rg_1_ (**70**), Rg_2_ (**71**), notoginsenoside R_2_ (**77**) and pseudoginsenoside F_11_ (**140**) from rhizomes of *P. japonicus* collected in Yunnan, China. The dammarane saponins were found to be significantly different from those of Chikusetsu-Ninjin and Himalayan *Panax* [59].

In 2011, further phytochemical investigation of the rhizomes of *P. japonicus* resulted in the isolation of two new dammarane-type triterpenoid saponins: yesanchinoside R_1_ (**99**) and R_2_ (**100**), together with one new natural product, 6′′′-*O*-acetyl-ginsenoside Re (**73**). In addition, 25 known compounds, including 23 triterpenoid saponins, *β*-sitosterol 3-*O*-*β*-D-glucopyranoside (**167**), and ecdysterone (**165**), were also identified. Six of the known saponins were reported for the first time from *P. japonicus* [60].

### *Panax japonicus* C. A. Meyer var. *major* (Burk.) Wu et Feng

As one of the Chinese *Panax* spp., *P. japonicus* var. *major* grows from Tibet to Yunnan at altitudes of 2500–4500 m, and the internodes of its long creeping rhizomes are elongated and slender, being distinguished from those of *P. japonicus*, which has short and thick internodes. The rhizomes of this plant, a Chinese herbal medicine named Zu-Tziseng, have been traditionally used as antitussive, expectorant, hemostatic and analgesic [2].

In 1982, J. Zhou and T. R. Yang, in cooperation with Hiroshima University of Japan, isolated two new dammarane-type saponins, majonoside R_1_ (**135**) and R_2_ (**136**), two known oleanolic acid saponins, chikusetsusaponin IVa (**153**) and V (**157**), together with two dammarane saponins, ginsenoside Rd (**11**) and notoginsenoside R_2_ (**77**) from rhizomes of *P. japonicus* var. *major* collected in Yunnan, China [61].

In 1984, four dammarane saponins including ginsenoside Rd (**11**), Rb_3_ (**9**), Rb_1_ (**7**) and Rc (**10**) were isolated from leaves of *P. japonicus*, which resembled constituents in the aerial parts, and were significantly different with those in roots and rhizomes [62].

From 1987 to 1989, seven saponins were isolated from the rhizomes of *P. japonicus* collected in Qinling Mountain, and a comparison of saponin constituents of this varieties collected in Qinling Mountain (Shaanxi) and Hengduan Mountains (Yunnan) was provided. It has been proved that saponins of oleanane type were main constituents and those of dammarane type were minor constituents [63]. Furthermore, a series of damarane type saponins including six new saponins named majoroside F_1_–F_6_ (**33**–**36, 137**–**138**), were isolated from the leaves of *P. japonicus* [64, 65].

### *Panax zingiberensis* Wu et Feng

*Panax zingiberensis*, a ginger-shaped perennial herbal plant of *Panax* spp., 20–60 cm tall, is a unique medicinal resource originated from southern Yunnan. It is often found in shelters under limestone evergreen broad-leaved forests, where is cool and humid with the average annual temperature about 17 °C. The rhizome of the root is lumpy, and it is used for the treatment of bruises, swelling, fractures, functional uterine bleeding and traumatic bleeding, as well as to promote the blood circulation.

In 1984, six triterpenoid saponins were isolated from the rhizomes of *P. zingiberensis* collected from Yunnan, China. Namely ginsenoside R_0_ (**157**), Rg_1_ (**70**), Rh_1_ (**72**), chikusetsusaponin IV (**159**) and IVa (**153**), together with the zingibroside R_1_ (**156**) [66].

### *Panax japonicus* C. A. Meyer var. *angustifolius* (Burk.) Chen et Chu

*Panax japonicus* var. *angustifolius*, a variety of *P. japonicus*, is mainly cultivated in western Yunnan and used as a folk medicine to promote blood circulation, help relieving pain and removing the phlegm. In 1985, 10 triterpenoid saponins were isolated from the rhizome of *P. japonicus*, and identified as ginsenoside R_0_ (**157**), Rd (**11**), Rg_1_ (**70**), Rh_1_ (**72**), notoginsenoside R_1_ (**76**), chikusetsusaponin IV (**159**), IVa (**153**), zingibroside R_1_ (**156**), oleanolic acid 28-*O*-*β*-D-glucoside (**151**) and oleanolic acid 3-*O*-*β*-D-glucoronoside (**152**), respectively. It is considered that there is a close relationship between var. *angustifolius* with *P. japonicus* and var. *major*, as their saponin constituents are similar. Oleane-type pentacyclic triterpenoid ginsenoside R_0_ (**157**), chikusetsusaponin IV (**159**) and IVa (**153**) are the main saponins in these plants, while they are in small amounts in dammarane type tetracyclic triterpenoid saponins [67].

### *Panax stipuleanatus* Tsai et Feng

*Panax stipuleanatus*, also known as “wild San-chi”, “Xiang-ci” and “slub San-chi”, is an herbal plant of the *Panax* genus in Araliaceae family. It is cultivated in Maguan, Malipo, Hekou and Pingbian, southeastern Yunnan, usually grows in the tropical seasonal rain forests at latitude of 1100–1700 m. The rhizomes have the effect of dispersing phlegm, relieving pain, stopping bleeding and nourishing. The main aglycone, oleanolic acid, panaxadiol and panaxatriol were once isolated from their crude saponin hydrolysates. In 1975, Zhou at el. isolated glycoside oleanolic acid and minor amount of panaxatriol and panaxadiol from the hydrolyzed products of saponins in *P. stipuleanatus* [3]. In 1985, C.R. Yang et al. isolated two oleanolic saponins, named as stipuleanoid R_1_ (**163**) and R_2_ (**164**), from the rhizome of *P. stipuleanatus* [68].

### *Panax japonicus* var. *bipinnatifidus* (Seem.) Wu et Feng

*Panax japonicus* var. *bipinnatifidus*, also known as “lump San-chi”, is located in the mountainous area of China, from the Northwest to the Southwest, with relatively high altitude and latitude in comparison with other species in the genus of *Panax*. In the area of Qinling Mountains, Shaanxi Province, it mainly grows in wet coniferous forests in the South and North Slope at an altitude of 2100–2900 m. The root has been used as a folk medicine, with effects of clearing away heat and toxic material, promoting digestion, activating blood circulation to remove blood fatigue, strengthening and nourishing [3].

In 1988, ten saponins were isolated from the rhizome of *P. japonicus* var. *bipinnatifidus*, collected in Qinling Mountain (Shaanxi, China), namely chikusetsusaponin V (**157**), IV (**159**), IVa (**153**), zingibroside R_1_ (**156**), ginsenoside Rb_1_ (**7**), Rd (**11**), Re (**146**), Rg_1_ (**70**), Rg_2_ (**71**) and 24(*S*)-pseudoginsenoside F_11_ (**140**), respectively. Their taxonomic significance were also discussed [69]. After that, two new dammarane type saponins bipinnatifidusoside F_1_ (XII) (**43**) and F_2_ (XIII) (**44**), along with eleven known saponins were further found from the dried leaves of *P. japonicus* var. *bipinnatifidus*, collected in Range of Qinling Mountains in China [70].

## Conclusions and Future Perspectives

Based on plant morphology, chemical composition and geographical distribution, the systematic evolution of *Panax* species was firstly discussed by the scholars in KIB, CAS, to have proposed a new classification system. Moreover, by using various phytochemical purification and structural identification techniques, the components and pharmacological activities of nine species in the genus *Panax* were investigated.

Among them, the chemical constituents of *P. notoginseng* were systematically studied, and dozens of compounds, mainly saponins were isolated and identified from different parts of *P. notoginseng*. The products collected from chemical, physical and biological transformation process of saponins in *P. notoginseng* were investigated as well. So far, nearly 286 compounds were reported from *P. notoginseng* [35–37, 71], 159 of which have been identified by KIB, CAS. Furthermore, the chemical constituents of *P. zingiberensis*, *P. japonicus* var. *angustifolius*, *P. stipuleanatus* and *P. japonicus* var. *bipinnatifidus* have only been studied by scholars in KIB, CAS.

At present, researches related to *Panax* species in KIB, CAS are mainly focused on the species of *P. notoginseng*, particularly for the secondary metabolites of its rhizospheric microbes and endophyte, and the transformation of saponins under various conditions. The isolated compounds from microbes and plant itself have also been studied for its interactions with the rhizospheric microorganisms, and effects on the seeds and plants of *P. notoginseng* as well as various crops. At the same time, many attentions will be paid to the difficulties and challenges faced by *P. notoginseng* in continuous planting and cultivation, under the multidisciplinary collaborative research.
